# Poster 1014: Omalizumab efficiency in patients with allergic rhinitis and chronic sinusitis

**DOI:** 10.1186/1939-4551-7-S1-P9

**Published:** 2014-02-03

**Authors:** Sandra Nora Gonzalez-Diaz, Lorena Rangel-Garza

**Affiliations:** 1 Universidad Autonoma de Nuevo Leon, Hospital Universitario, Monterrey, Mexico; 2 Universidad Autonoma de Nuevo Leon, Hospital Universitario "Dr. Jose E. Gonzalez" UANL, Monterrey, Mexico

## Background

Allergic rhinitis (AR) is a very common chronic respiratory disorder and has also been shown to negatively impact quality of life with many comorbiliditys like Chronic rhinosinusitis; There is evidence that IgE antibodies play a role in chronic sinusitis. Investigations have shown that total IgE levels correlate with the severity of sinusitis, as assessed by CT scan. Allergies occur more frequently in patients with chronic sinusitis than in the general population. Omalizumab has shown to be effective for AR in allergen challenge studies and clinical trials. Multiple randomized, double-blind, placebo-controlled studies have shown efficacy of omalizumab in seasonal and perennial AR.

## Methods

All patients form private consult with AR who also had chronic rhinosinusitis in which symptoms were evaluated using the analog visual scale (VAS) at the beginning of their medical treatment. All subjects included in this study had skin prick positive tests to multiple aeroallergens and food without improvement of their symptoms after one year of specific subcutaneous immunotherapy to whom posterior Omalizumab of 150 to 300 mg was administrated every 4 weeks at least for one year and who were evaluated again using the analog visual scale at the end of their treatment.

## Results

11 patients were included, 6 females, 1 of them with rhinosinusitis and nasal polyps (CRSwNP) and 5 males. Ages were from 7 to 71 years (34.5 years average). The analog visual scale at the beginning had and average of 4.5 points and after a year with Omalizumab scores increased to an average of 8.3 points with an increase of 3.8 points. The only patient with chronic rhinosinusitis and nasal polyps (CRSwNP) presented polyps remission with an improvement of life quality. None of the patients presented severe adverse reactions from the application of Omalizumab.

## Conclusions

Clinical improvement was found and omalizumab could be considered of benefit in patients with chronic rhinosinusitis who also has proved to be secured

